# Harnessing stem cell-derived exosomes: a promising cell-free approach for spinal cord injury

**DOI:** 10.1186/s13287-025-04296-4

**Published:** 2025-04-17

**Authors:** Miaoman Lin, Farzaneh Alimerzaloo, Xingjin Wang, Obada Alhalabi, Sandro M. Krieg, Thomas Skutella, Alexander Younsi

**Affiliations:** 1 Department of Neurosurgery, Heidelberg University Hospital, Im Neuenheimer Feld 400, 69120 Heidelberg, Germany; 2 Medical Faculty, Heidelberg University, Heidelberg, Germany; 3 Department of Neuroanatomy, Institute of Anatomy and Cell Biology, Heidelberg University, Heidelberg, Germany

## Abstract

Spinal cord injury (SCI) is a severe injury to the central nervous system that often results in permanent neurological dysfunction. Current treatments have limited efficacy and face challenges in restoring neurological function after injury. Recently, stem cell-derived exosomes have gained attention as an experimental treatment for SCI due to their unique properties, including superior biocompatibility, minimal immunogenicity and non-tumorigenicity. With their potential as a cell-free therapy, exosomes promote SCI repair by enhancing nerve regeneration, reducing inflammation and stabilizing the blood-spinal cord barrier. This review summarizes advances in stem cell-derived exosome research for SCI over the past years, focusing on their mechanisms and future prospects. Despite their promising therapeutic potential, clinical translation remains challenging due to standardization of exosome isolation protocols, compositional consistency and long-term safety profiles that require further investigation.

## Introduction

Spinal cord injury (SCI) is a devastating traumatic disease of the central nervous system (CNS), typically caused by mechanical damage resulting from traffic accidents, sports injuries, falls, and self-inflicted violence [1, 2]. The pathophysiology of the secondary injury after SCI is highly complex, involving, i.e., spinal cord ischemia, edema, oxidative stress, inflammatory responses, neuronal apoptosis and demyelination, and excitatory amino acid and ionic imbalances [3, 4]. SCI often results in permanent motor, sensory, and autonomic dysfunction, including paralysis, sensory abnormalities, spasticity, and pain, as well as cardiovascular, bowel, bladder, or sexual dysfunction. High-level spinal cord injuries can lead to life-threatening conditions such as respiratory and cardiac arrest [1, 3]. Current treatment strategies for SCI primarily encompass decompression and stabilization surgery, supportive medical care, and physical rehabilitation therapy [5]. However, their overall efficacy is quite limited, and reversing the neurological damage caused by the injury remains challenging [6]. This significantly diminishes patients’ quality of life and places a heavy economic and psychological burden on families, healthcare systems, and society [1]. As a result, there is a pressing need to discover new and more effective treatment options.

Stem cells—with their self-renewal capacity and ability to differentiate into multiple cell types—offer a promising avenue for SCI treatment by potentially repairing damaged tissues through differentiation into neurons or supportive cells. Consequently, researchers are increasingly focusing on stem cell therapies [7–9]. Among the various types of stem cells studied for transplantation, several have demonstrated early progress in cell replacement therapy for SCI [3, 6, 10–12]. Preclinical and phase I clinical studies suggest that stem cell therapies can promote recovery from neurodegenerative diseases and neurological injuries through neuroprotection, immunomodulation, neuronal relay formation, and remyelination [8]. However, the efficacy and safety of stem cell transplantation remain under-evaluated, with several challenges yet to be addressed [13]. In this context, exosomes, nano-sized vesicles secreted by stem cells, have emerged as a safer and more controllable alternative. Exosomes not only carry biologically active molecules like proteins, microRNAs (miRNA, miR), and messenger RNAs (mRNA) but also exhibit enhanced circulatory stability, blood-spinal cord barrier (BSCB) penetrability, and reduced immunogenicity compared to parental cells [14, 15]. Recent studies demonstrate that stem cell-derived exosomes promote SCI repair through neural regeneration [16], anti-inflammation [17], immune modulation [18], and BSCB repair [19]. These findings suggest that exosomes have great potential in SCI treatment. This narrative review categorizes stem cell-derived exosomes by their cellular origin and systematically analyzes their repair mechanisms, providing a framework for future translational research.

## Mechanisms of SCI

**Fig. 1:**
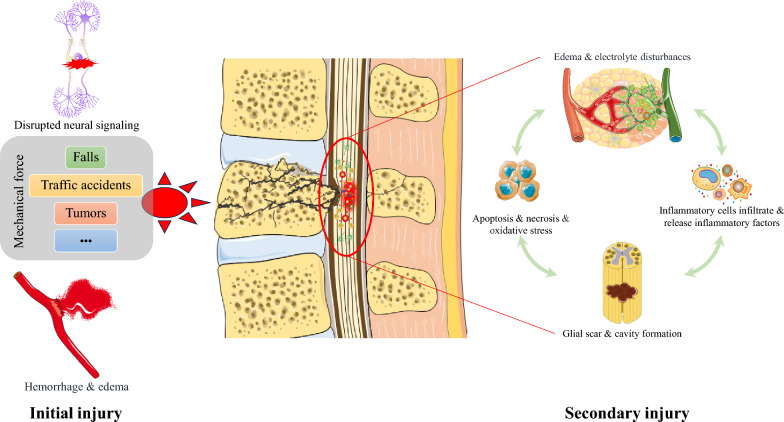
Schematic diagram of spinal cord injury mechanisms

The pathophysiologic mechanisms of SCI are typically divided into two phases: the initial injury and secondary injury cascades, each involving complex biological processes (Fig. 1) [3].

### Initial injury

The initial injury is usually directly caused by external trauma (e.g., compression, contusion, or puncture), which results in mechanical damage to the spinal cord tissue. The main effects include neuronal death by mechanical force, which extends to the axons within the spinal cord and leads to loss of conduction function [20, 21]. Damage to blood vessels at the site of injury, resulting in local hemorrhage and disrupting blood supply, thus triggering edema and ischemia [22]. Mechanical compression of the spinal cord due to spinal instability or fractures results in localized edema and hematoma formation, which further aggravates the injury [3].

### Secondary injury

Secondary injury cascades are more complex pathological processes that occur after the initial trauma, usually within hours to weeks, leading to further neuronal death and loss of function. The mechanisms involved include an inflammatory response triggered by immune cells such as neutrophils, macrophages, and T cells that release inflammatory factors [e.g., Tumor necrosis factor-α (TNF-α), Interleukin-1β (IL-1β), IL-6], which may help clear necrotic tissue, but also exacerbates nerve damage [23]. Oxidative stress is mediated by free radicals and reactive oxygen species (ROS) that are generated after the injury and might damage neurons and cell membranes, which further exacerbates spinal cord degradation by destroying cellular structures and inducing apoptosis [21]. Apoptosis itself results in programmed cell death of neurons and supportive cells such as oligodendrocytes, further reducing the repair and regenerative capacity of the spinal cord [20]. Calcium overload is a result of dysregulated intracellular calcium levels that activate calcium-dependent enzymes, leading to the breakdown of structural proteins in neurons, further compromising neuronal function and cell membrane integrity [21]. Vascular damage and decreased blood supply after injury cause an ischemic and hypoxic environment, affecting the normal function of the spinal cord and impeding the regenerative process [22]. Glial scarring at the injury site, mediated by proliferating astrocytes that physically block axonal regeneration and repair, hinders nerve recovery after injury [24].

Given the combination of primary and secondary injuries, there is a significant loss of neurons and glial cells in the spinal cord, leading to long-term sensory, motor, and autonomic dysfunction. These impairments are irreversible and severely impact the quality of life of affected patients [1, 3, 21]. In summary, the pathophysiological mechanisms of SCI are highly complex, involving mechanical injury, inflammation, oxidative stress, cell death, and apoptosis. This complexity poses a significant challenge for effective treatment. Notably, the temporal-spatial dynamics of secondary injury create therapeutic windows that are often underutilized in current approaches. For instance, the early inflammatory phase (0–72 h) may require different intervention strategies compared to the subacute remodeling phase (1–4 weeks). This temporal specificity highlights the need for therapeutic agents with dynamic modulatory capacities—a feature potentially achievable through engineered exosomes carrying stage-specific cargo [90].

## Stem cell and exosome characterization

### Properties and limitations of stem cells

Stem cells are classified into various types, with the stem cells currently used in therapeutic research for SCI including neural stem cells (NSC), mesenchymal stem cells (MSC), embryonic stem cells (ESC), induced pluripotent stem cells (iPSC), and dental pulp stem cell (DPSC), each with distinct regenerative properties [3, 6, 10–12, 25, 26]. However, stem cell therapy faces several significant challenges, such as immune rejection, tumorigenic risks, the low survival rate of transplanted cells, and the complexity of the technique itself [7, 9, 10, 12, 21, 25]. As a result, there is an urgent need to explore safer, more controllable, and widely applicable therapeutic strategies.

### Advantages of exosomes

**Fig. 2:**
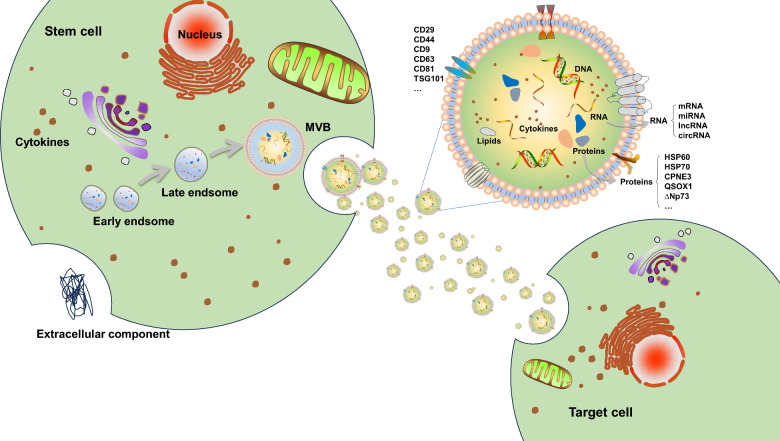
The biogenesis process of exosomes

Exosomes are membrane-bound vesicles, ranging from 30 to 150 nm in diameter, released by cells through exocytosis; exosome biogenesis is highly intricate (see Fig. 2 for details). Exosomes are rich in proteins, lipids, mRNAs, miRNAs, and many other biologically active molecules [27]. Compared with the direct use of stem cells, exosomes offer several advantages for treating SCI, including high biocompatibility, circulatory stability, low immunogenicity, non-carcinogenicity, editability, capacity to pass the BSCB, and ease of storage [8, 28, 29]. Stem cell-derived exosomes have gradually become a hotspot for SCI therapy research due to their efficiency, safety, and user-friendly properties.

## Exosomes derived from different types of stem cells for SCI treatment

**Fig. 3:**
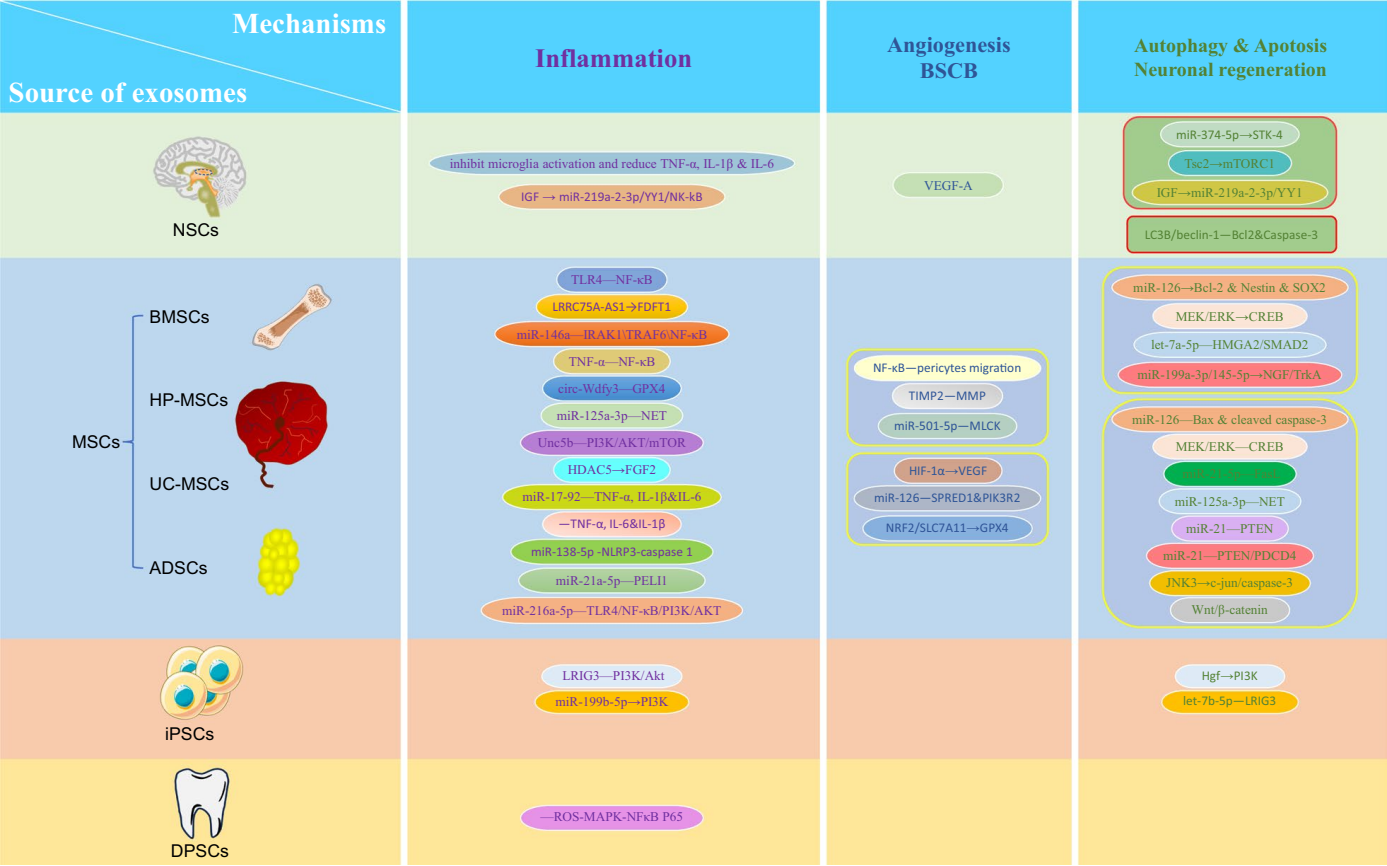
Comparison of the mechanisms of exosomes derived from different cell types for spinal cord injury

To systematically evaluate the therapeutic potential of stem cell-derived exosomes in SCI, we categorize them based on their cellular origin (NSC, MSC, iPSC, and DPSC) and analyze their distinct repair mechanisms (Fig. 3).

### NSC-derived exosomes

**Fig. 4:**
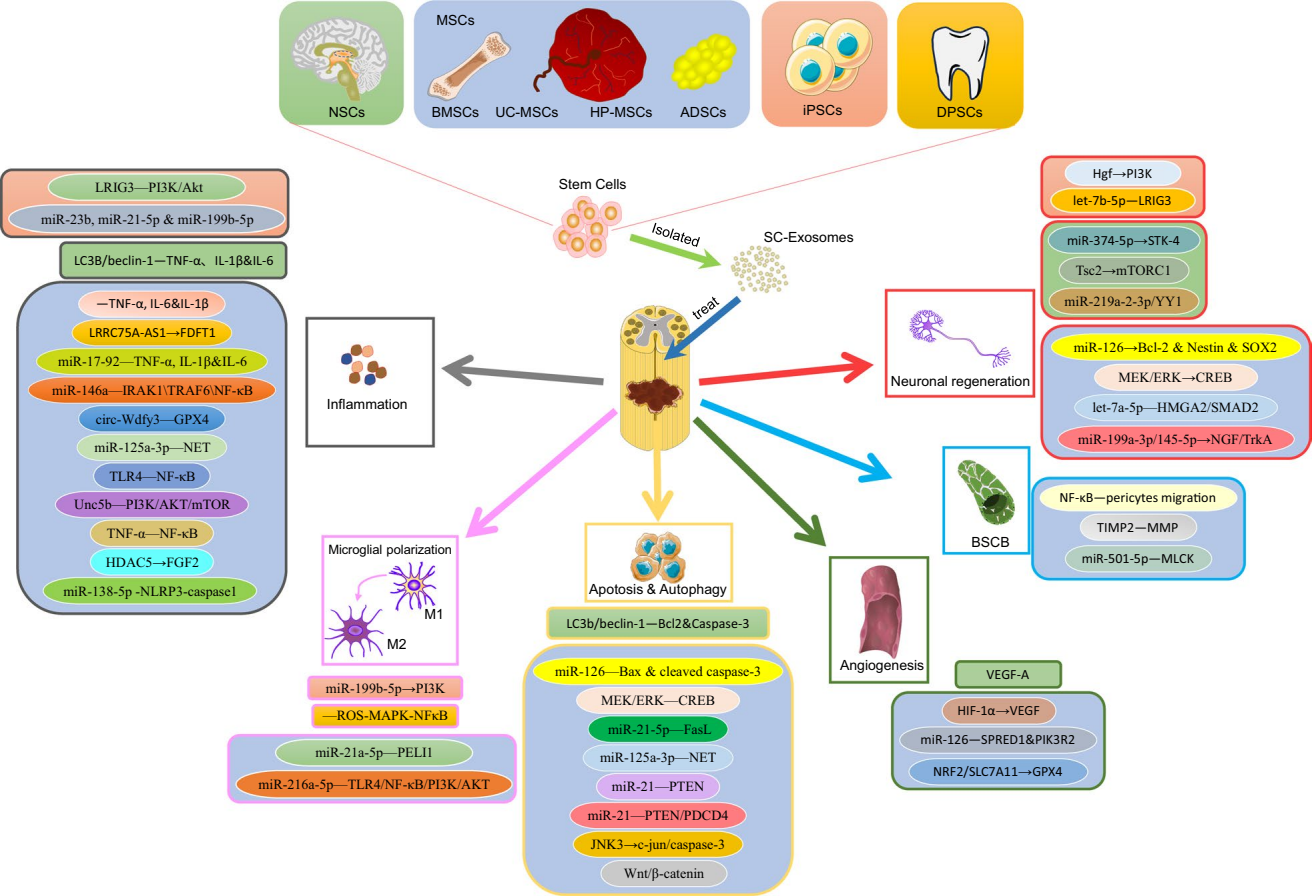
Origin of exosomes and their related mechanisms in the treatment of spinal cord injury

NSC can differentiate into neurons, astrocytes, and oligodendrocytes [30]. Localized transplantation of NSCs has also been shown to have a therapeutic effect on SCI, the effect that may be related to cytokines such as brain-derived neurotrophic factor (BDNF), vascular endothelial growth factor (VEGF), or nerve growth factor (NGF); however, given that implantation efficiencies are very low (< 5%) and that transplants typically result in a severe inflammatory immune microenvironment, exogenous NSC transplantation exists controversial [31]。 NSC-derived exosomes are enriched in NGF, BDNF, and various miRNAs [31]. These exosomes promote neuronal regeneration and improve functional recovery after SCI by preventing neuronal apoptosis and regulating autophagy, reducing inflammation, and promoting angiogenesis (Fig. 4 & Table 1), are emerging as a new research field.

**Table 1:** Research on the treatment of SCI with exosomes derived from stem cells

Year	Source of exosomes	SCI models	Administration route	Dose	Target(s)	Function(s)	References
2022	NSC	C57BL/6 mouse T10 contusion SCI	Tail vein	Unspecified (in 30 ul PBS for once)	miR-374-5p/STK-4 axis	Promote autophagy	[32]
2019	NSC	SD rat T10 contusion SCI	Tail vein	200 μg in 200 μL of PBS for once	LC3B & beclin-1	Promote autophagy	[33]
2024	NSC	C57BL/6 mouse T10 contusion SCI	/ (Single-cell RNA sequencing)	Unspecified	TSC2/EGF & MDK *Slc16a3*/VEGF & MIF	Increase autophagic flux	[34]
2019	NSC	SD rat T10 contusion SCI	Tail vein	100 μg in 0.5 mL of PBS for once	miR-219a-2-3p-YY1/NF-κB	Suppress neuroinflammation and apoptosis, enhance pro-neuroregenerative capacities	[35]
2020	NSC	C57BL/6 mouse T10 contusive SCI	Tail vein	200 µg in 100 µL PBS at 30 min after SCI tills on 3, 6, 9, and 12 d post-SCI, respectively	VEGF-A	Promote microvascular regeneration, tissue healing, and reduce tissue cavity formation	[36]
2024	hAMSC	SD rat T10 contusion SCI	Tail vein	200 μg in 200 µl PBS for once	reduce ROS and inflammatory cytokines levels, including TNF-a, IL-6, and IL-1β	Promote the functional recovery of TSCI rats by Reduce neuroinflammation, cell apoptosis, astrogliosis, and lesion volume, protect the BSCB, and enhance angiogenesis and axonal regeneration	[55]
2024	ADSC	Hemin induced mouse microglia BV2 post-SCI inflammation cell model	Bioinformatics prediction	Unspecified (with a concentration of 3 × 10^9^ particles/ml for co-culture)	LRRC75A-AS1/FDFT1	Inhibit inflammation	[17]
2024	BMSC	Rat T10 contusion SCI	Tail vein	100 μg in 0.5 mL PBS for 7 consecutive days	inhibit pro-inflammatory factors (TNF-α, IL-1β, and IL-6)	Modulate neuronal apoptosis and inflammation	[57]
2024	BMSC	SD rat T10 contusion SCI	Tail vein	Unspecified (in 500ul PBS for once)	miR-146a-IRAK1\TRAF6\NF-κB p65	Reduce inflammatory factors and oxidative stress	[43]
2024	Hypoxia-preconditioned ADSC	C57BL/6 mouse T10 contusive SCI	Tail vein	200ug in 200ul PBS for once	circ-Wdfy3/miR-423-3p/GPX4	Reduce the levels of ROS and inflammatory cytokines	[50]
2024	AMSC	SD rat T6/7 contusion SCI	Tail vein	100 μg in 1 mL of PBS for 3 consecutive days	miR-125a-3p	Diminish inflammatory Neutrophil extracellular trap formation	[54]
2024	UCMSC	Rat T10 contusion SCI	Tail vein	100 ug in 0.5 mL PBS for once	NF-κB/MAPK	Reduce the inflammatory response	[49]
2023	hPMSC	SD rat T9 contusion SCI	Tail vein	200 ug in 200ul PBS for once	unspecified	Ameliorate histological changes, reduce inflammation, apoptosis, and gliosis, modulate oxidative stress (in combination with Hyperbaric Oxygen)	[87]
2023	BMSC	SD rat contusion SCI	Tail vein	200 μg in 500 μL PBS for once	miR-216a-5p-TLR4/NF-κB	Attenuate neuronal injury and microglia-mediated inflammation	[45]
2021	BMSC	SD rat T9/10 contusion SCI	Tail vein	200 μg in 0.5 mL PBS for once	miR-145-5p-TLR4/NF-κB	Reduce inflammation	[46]
2021	BMSC	SD rat T10 contusion SCI	Tail vein	Unspecified (200 µL of exosomes for 7 consecutive days)	TLR4/MyD88/NF-κB	Reduce apoptosis and inflammatory response	[47]
2023	hUCMSC	SD rat T10 contusion SCI	Injected into the surface of the spinal cord	unspecified	miR-138-5p-NLRP3-caspase1 & Nrf2-keap1	Reduce the inflammatory response	[48]
2023	hUCMSC	SD rat spinal cord ischemia/reperfusion model	Intrathecally injection	20 μg exosomes in 10 μL PBS for once	miR-146a-5p-TLR4/NF-κB	Alleviate spinal cord injury and inflammatory cytokines	[44]
2023	BMSC	SD rat T10 contusion SCI	Tail vein	200 μg in 500 μl PBS for once	miR-137	Enhance neuron viability while reducing tissue injury and diminish tissue inflammation	[59]
2023	BMSC	SD rat T10 contusion SCI	Tail vein	200 μg in 500 μl PBS at 1h, 7, 14 d after SCI	miR-146a	Promote neuron viability while repressing apoptosis and inflammation	[58]
2023	BMSC	SD rat T10 contusion SCI	Tail vein	100 μg in 0.5 mL of PBS (equivalent to 1 × 10^10^ particles) for consecutive 5 days	netrin-1- Unc5b/PI3K/AKT/mTOR	Mitigate the inflammatory response, reduce pyroptosis, and promote the growth of axons	[90]
2022	hUCMSC	C57BL/6J T11&12 contusion SCI	Caudal vein	50 μg in 200 μL of PBS for once	TNF-α/NF-κB	Attenuate the inflammation and tissue damage	[91]
2022	BMSC	SD rat T10 contusion SCI	Tail vein	100 μg in 0.5 mL PBS for consecutive 7 days	miR-9-5p-HDAC5/FGF2	Inhibit LPS-induced apoptosis, inflammation, and ER stress in vitro. improve locomotor ability and alleviate histopathological damage and neuronal apoptosis in vivo	[51]
2019	hUCMSC	F344-rat T8 contusion SCI	Tail vein	100 μL of Ringer-lactate containing the hUCMSC-EVs secreted by 10^6^ hUCMSCs twice	quench the expression of pro-inflammatory cytokines (IL-1β and IL-6)	Anti-inflammatory and anti-scarring activities	[56]
2024	BMSC	C57BL/6J mouseT8 contusive SCI	Intrathecal injection	100 μg in 5 μL PBS for once	miR-21a-5p/PELI1	Enhance autophagy and suppression of pyroptosis in macrophage/microglia, and attenuate the inflammatory response	[53]
2020	hypoxia-preconditioned BMSC	C57BL/6 mouse T10 contusive SCI	Tail vein	200 μg in 200 μL PBS for once	miR-216a-5p-TLR4/NF-κB & PI3K/AKT	Shift microglia from the M1 phenotype to the M2 phenotype	[52]
2020	placental mesenchymal stem cell	mouse T10 contusive SCI	The epicenter of the SCI	Unspecified (with a concentration of 200 μg/μL) for once	unspecified	Promote endothelial cells formatted capillary-like structures in vitro and stimulate neoangiogenesis in vivo	[61]
2022	Hypoxia-pretreated hUCMSC	transection SCI	Transplanted to the spinal lesion	100 μg in 20 μL PBS (Encapsulation of exosomes in the hydrogel)	HIF-1α	Enhance angiogenesis and nerve regeneration	[62]
2020	BMSC	SD rat T10 contusion SCI	Tail vein	100 μg in 0.5 mL of PBS for once	miR-126-SPRED1 & PIK3R2	Promote angiogenesis and neurogenesis and attenuate apoptosis	[63]
2024	ADSC	SD rat T10 contusion SCI	Tail vein	100 µg in 1 ml PBS with DMSO for once	NRF2/SLC7A11/GPX4	Protect endothelial cells and promote their regeneration by inhibiting ferroptosis	[64]
2019	BMSC	SD rat T10 contusion SCI	Tail vein	200 μL of EV (200 μg/mL) for twice	NF-κB p65	Inhibit pericyte migration and improve the integrity Improvement of the BSCB	[65]
2021	hBMSC	SD rat T9 contusion SCI	Subcutaneous injection	100 μg in 100 μl of PBS, once a day for 1 week	TIMP2/MMP	Attenuate BSCB disruption	[66]
2023	UCMSC	Mouse T 10 Contusive SCI	Intranasally administered	1 mg/kg at 3 h and 3 and 6 days after SCI	RGD-CD146CD271-miR-501-5p/MLCK	Reduce the disruption of tight junctions, stabilize the BSCB	[19]
2022	BMSC	SD rat T10 contusion SCI	Unspecified	200 μg in 200 μl of PBS for once	Wnt/β-catenin	Reduce cell apoptosis and potentially reduce the activation of astrocytes and microglia cells	[92]
2019	BMSC	Wistar rat T10 hemisection SCI	Tail vein	100 μg in 0.5 mL of PBS for once	microRNA-21-5p/FasL	Attenuate apoptosis and improve neurological function	[93]
2019	hMSC	SD rat T9/10 contusion SCI	Intravenous injection	Unspecified	miR-21 and miR-19b -PTEN	Regulate the apoptosis and differentiation of neuron cells	[94]
2019	MSC	SD rat T9/10 contusive SCI	i.v. injection	Unspecified	MiR-21-PTEN/PDCD4	Inhibit the cell apoptosis of neurons	[95]
2022	hypoxia-induced ADSC	SD rat SCI	Tail vein	Unspecified	miR-499-5p-JNK3/c-Jun/MAPK10	Inhibit neuronal apoptosis	[96]
2021	hPMSC	SD rat T11 contusive SCI	Tail vein	50 μg in 100 μl PBS for twice	MEK/ERK/CREB	Modulate endogenous NPCs and enhance neurogenesis	[68]
2021	UCMSC	SD rat T10 contusive SCI	i.v. injection	200 μg for twice	miR-199a-3p/145-5p-NGF/TrkA	Promote neuronal differentiation and neurite outgrowth	[69]
2022	BMSC	Wistar rat T10 contusive SCI	Intrathecal injection	polyethylene catheter continuous injection of 25 μl BMSC-EV-injection, 50 nmol let-7a-5p for 3 days	let-7a-5p-HMGA2/SMAD2	Mediate the differentiation of NSCs and promotes the regeneration of neurons and their neurite outgrowth in glial scars	[70]
2022	iPSC	C57BL/6 mouse T11/12 contusive SCI	Tail vein	unspecified (200 μl for 3 days)	miR-199b-5p-Hgf /PI3K	Shift the polarization from M1 macrophage to M2 phenotype and regulate related inflammatory factors expression	[71]
2024	iPSC	C57BL/6 mouse T8 contusive SCI	Intrathecal injection	unspecified (with a concentration of 20 µg/µL) for once	let-7b-5p-LRIG3/PI3K/Akt	Modulate microglial/macrophage pyroptosis, promote axonal growth	[73]
2024	iPSC	C57BL/6 mouse T11/12 contusive SCI	i.v. injection	30 µg for once	miR-23b, miR-21-5p & miR-199b-5p	Reduce inflammatory cell infiltration and apoptosis	[72]
2022	DPSC	C57BL/6 mouse T11/12 contusive SCI	Tail vein	200 μg in 100 μl PBS for once	ROS-MAPK-NFκB P65	Reduce macrophage M1 polarization, attenuate the inflammatory response, and reduce neurological impairment	[26]

NSC = neural stem cells; SCI = spinal cord injury; PBS = phosphate buffered saline; miRNA, miR = microRNAs; Tsc2 = tuberous sclerosis 2; MDK = midkine; Slc16a3 = solute carrier family 16 member 3; VEGF = vascular endothelial growth factor; MIF = macrophage migration inhibitory factor; NF-κB = transcription factor kappa B; hAMSC = human amniotic mesenchymal stem cells; ROS = free radicals and reactive oxygen species; TNF-α = tumor necrosis factor-α; IL = Interleukin; ADSC = adipose-derived mesenchymal stem cells; BMSC = bone marrow mesenchymal stem cells; IRAK1 = interleukin-1 receptor-associated kinase 1; GPX4 = glutathione peroxidase 4; MAPK = mitogen-activated protein kinase; UCMSC = umbilical cord mesenchymal stem cells; hPMSC = human placental mesenchymal stem cells; TLR4 = toll-like receptor 4; MyD88 = myeloid differentiation factor 88; PI3K = phosphatidylinositol-3-kinase; PKB or AKT = protein kinase B; mTOR = mammalian target of the rapamycin; HDAC5 = histone deacetylase 5; FGF2 = fibroblast growth factor 2; LPS = Lipopolysaccharide; HIF-1α = hypoxia-inducible factor 1-alpha; SPRED1 = Sprouty-related EVH1 domain-containing protein 1; NRF2 = nuclear factor erythroid-2-related factor 2; SLC7A11 = solute carrier family 7 member 11; BSCB = blood-spinal cord barrier; TIMP2 = tissue inhibitors of matrix metalloproteinase 2; MMP = matrix metalloproteinase; RGD = Arg-Gly-Asp; MLCK = myosin light chain kinase; FasL = fas ligand; PTEN = phosphatase and tensin homolog; PDCD4 = programmed cell death 4 protein; MEK = mitogen-activated protein kinase kinase; ERK = extracellular signal-regulated kinases; CREB = cAMP response element binding; NGF = Neuronal growth factor; TrkA = Tropomyosin receptor kinase A; EV = extracellular vesicles; HMGA2 = high-mobility group A 2; iPSC = induced pluripotent stem cells; Hgf = hepatocyte growth factor; DPSC = dental pulp stem cell

#### Apoptosis and autophagy regulation

Recent studies have focused on NSC-derived exosomes’ role in regulating apoptosis and autophagy. For instance, NSC-derived exosomes can activate autophagy by targeting the miR-374-5p/STK-4 axis, which inhibits neuronal apoptosis and aids in SCI repair. miR-374-5p regulates autophagy-related genes by modulating STK-4 activity, enhancing autophagy to clear damaged cells and maintain neuron survival [32]. However, these findings are largely based on preclinical models, and their clinical relevance remains uncertain. Moreover, NSC-derived exosomes can increase the expression of autophagy markers like LC3B and beclin-1, facilitating autophagosome formation, reducing SCI damage, and decreasing pro-apoptotic proteins like Bax and caspase-3, while increasing anti-apoptotic proteins like Bcl-2, further mitigating neuronal apoptosis [33]. A recent study utilizing single-cell RNA sequencing and transcriptomic analysis verified that NSC originates from ependymal cells and identified increased expression of Tsc2(compared with the sham group, *p* = 0.0298), a hub gene in the competing endogenous RNAs (ceRNA) network. The study further explained that following necrotic apoptosis, NSC-derived exosomes can reduce the damage of spinal cord tissue by inhibiting mTORC1 expression, thereby increasing autophagic flux [34]. The balance between autophagy activation and apoptosis inhibition requires further investigation. Future studies should clarify the precise mechanisms, potential side effects, and feasibility of clinical application.

#### Neuroinflammation suppression

NSC-derived exosomes also reduce pro-inflammatory cytokines such as TNF-α, IL-1β, and IL-6 and inhibit activation of microglia, thus reducing the inflammatory response [33]. Additionally, Ma et al. [35] found that cell viability was higher and axons were longer in IGF-Exo group's PC12 cells than in the injury model group or Nor-Exo group (*p* < 0.05), which demonstrated that NSC-derived exosomes exposed to IGF-1 could partially suppress neuroinflammation and apoptosis while enhancing pro-neuroregenerative capacities via a miR-219a-2-3p/YY1/NK-kB-dependent mechanism. However, while this finding is promising, the mechanisms involved remain complex and context-dependent, and the long-term effects of exosome-based therapies on neuroinflammation in humans are still unknown. Additionally, the potential off-target effects of targets inhibition and the incomplete understanding of pathway warrant further investigation before clinical applications can be considered.

#### Angiogenesis promotion

A groundbreaking study, for the first time, discovered that NSC-derived exosomes are enriched with vascular endothelial growth factor (VEGF) A, which promotes microvascular regeneration and tissue healing and reduces tissue cavity formation thus restoring neurological functions following SCI (BMS score in the NSCs^Con shRNA^-Exos groups and control groups on 28 day = 4.30 ± 0.95 vs. 6.70 ± 1.25, *P* < 0.01) [36]. This indicated that NSC-derived exosomes may enhance SCI repair by promoting angiogenesis and fostering neural regeneration.

In conclusion, although NSC-derived exosomes show promising potential in SCI repair, their application remains primarily in basic research. The underlying mechanisms are diverse and vary from study to study, with no consensus yet on their therapeutic pathways or standardized use. Future research should focus on unravelling the precise molecular mechanisms of NSC-derived exosomes, identifying key bioactive components and exploring their interactions within the SCI microenvironment. Standardized protocols for exosome isolation, characterization and delivery need to be established to ensure reproducibility and efficacy. In addition, preclinical and clinical studies are essential to evaluate their safety, optimal dosage and therapeutic efficacy, paving the way for their eventual translation into clinical applications [37–39].

### MSC-derived exosomes

MSC-derived exosomes are one of the most widely studied exosome types due to their diverse sources (e.g., bone marrow, umbilical cord, adipose tissue, etc.) and robust immunomodulatory capabilities [40–42]. They promote SCI repair through three core mechanisms: anti-inflammation, angiogenesis/BSCB stabilization, and apoptosis inhibition/nerve regeneration (Fig. 4 & Table 1).

#### Anti-inflammatory effects

Inflammation plays a critical role in SCI, and the balance between pro-inflammatory and anti-inflammatory components within the local microenvironment of SCI is closely associated with post-injury prognosis. Modulating the microenvironment to suppress pro-inflammatory responses while promoting anti-inflammatory processes represents a key therapeutic strategy for SCI treatment.

The inflammatory cascade following SCI involves a complex interplay of signaling pathways and immune cells. MSC-derived exosomes counteract the inflammatory process through three main synergistic strategies:

##### Targeting key inflammatory pathways

The TLR4/NF-κB axis is a central driver of neuroinflammation. Bone marrow mesenchymal stem cell (BMSC)-derived exosomes loaded with miR-146a disrupt this pathway by inhibiting IRAK1/TRAF6/NF-κB p65, significantly reducing inflammatory factors in SCI rats (the levels of TNF-α, IL-1β and IL-6 in spinal cord tissues of the miR-146a-EXO group were lower than those of the control group at 1d, 3d, 1, 2 and 4 weeks after surgery, *p* < 0.05) [43]. This mechanism is corroborated by Wang et al., who employed miR-146a-5p-engineered human mbilical cord mesenchymal stem cell (hUCMSC)-exosomes to achieve similar TLR4/NF-κB suppression [44]. Other studies also found that miR-216a-5p or miR-145-5p carried by BMSC-derived exosomes inactivated the TLR4/NF-κB pathway in rats after SCI, which attenuated the inflammatory response and protected the spinal cord [45–47]. Beyond TLR4, UCMSC derived exosomes modified with miR-138-5p can reduce the inflammatory reaction of BV-2 cells through the NLRP3-caspase1 signaling pathway [48]. Notably, UCMSC-derived exosomes also can modulate inflammatory responses through the NF-κB/MAPK signaling pathway [49]. Another recent study demonstrated that hypoxia-preconditioned adipose-derived mesenchymal stem cells (ADSC) derived exosomes reduced the levels of ROS and inflammatory cytokines through the circ-Wdfy3/miR-423-3p/GPX4 signaling pathway [50]. BMSC derived exosomes loaded with miR-9-5p can inhibit LPS-induced inflammation by promoting FGF2 expression by inhibiting HDAC5-mediated deacetylation [51]. Another study found ADSC derived exosomes transfected with LRRC75A-AS1 upregulated the mRNA expression of FDFT1 and inhibited the inflammatory response after SCI via ceRNAs- and RNA-binding proteins-dependent pathways [17]. Although extensive research has focused on the anti-inflammatory effects mediated by the TLR4/NF-κB pathway, with additional studies exploring other pathways such as NLRP3-caspase1 and NF-κB/MAPK, these findings predominantly originate from animal-based models and require further validation for clinical translation.

##### Reprogramming the immune landscape

Microglia and macrophages dictate inflammatory progression following SCI. Hypoxia-preconditioned BMSC exosomes pivot microglia from pro-inflammatory M1 to anti-inflammatory M2 phenotypes via miR-216a-5p—a single miRNA that concurrently inhibits TLR4/NF-κB and activates PI3K/AKT pathways, in which alleviated the inflammation induced by M1 following SCI [52]. It was found that BMSC-derived exosomes mediate enhanced autophagy and focal death inhibition in macrophages/microglia through the miR-21a-5p/PELI1 axis and attenuate the immune cells related inflammatory response after SCI [53]. Beyond central immunity, peripheral neutrophils exacerbate secondary injury. Exosomes derived from human amniotic MSC (AMSC) carrying miR-125a-3p dismantle neutrophil extracellular traps, reducing myeloperoxidase activity and curbing neutrophil infiltration in the lesion immune micro-landscape [54]. AMSC-derived exosomes significantly decreased ROS, myeloperoxidase activity, and inflammatory cytokines (e.g., TNF-α, IL-6, and IL-1β) in the microenvironment, all of which contribute to neuronal protection [55]. After acute SCI, early intravenous application of hUCMSCs or hUCMSC-extracellular vesicles (EV) significantly dampened the accumulation of Iba1^+^ cells by ~ 25–30% in both gray and white matter, which significantly reduced the expression of pro-inflammatory cytokines in the spinal cord parenchyma and promoted the recovery of neurological function [56]. These studies collectively demonstrate exosomes’ capacity to modulate and reprogram the immune micro-landscape at injury sites. However, it is critical to note that these findings are derived from rodent models, and significant divergences may exist in human immune environments.

##### Other effects

BMSC-derived exosomes enriched with miR-17-92 significantly inhibited pro-inflammatory factors, including TNF-α, IL-1β, and IL-6 in spinal cord tissues after SCI and promoted nerve repair [57]. Other studies have also confirmed that miR-146a or miR-137 loaded in BMSC-derived exosomes similarly inhibited the expression of pro-inflammatory cytokines in the injured spinal cord tissues and enhanced locomotor activity and neuronal viability in SCI rats [58, 59]. All above demonstrate some anti-inflammatory effects through RNA-loaded exosomes, but the lack of clearly defined mechanisms limits their translational relevance for future clinical applications.

While most current research on MSC-derived exosomes for anti-inflammation are still in preclinical stages and lacks large-scale clinical validation, these findings highlight the significant potential of MSC-derived exosomes to mitigate SCI-induced inflammation and improve neurological recovery. Future research should prioritize clinical safety and efficacy studies to facilitate the translation of these therapies into clinical practice.

#### Angiogenesis and BSCB repair

Following SCI, vascular damage leads to ischemia, hemorrhage, and immune cell infiltration, which not only exacerbates the secondary injury but also further worsens neurological function. Thus, promoting angiogenesis is crucial for tissue survival and regeneration [60]. Research shows that MSC-derived exosomes significantly enhance both angiogenesis and functional recovery. For instance, exosomes derived from placental mesenchymal stem cells (PMSC) promoted endothelial cells formatted capillary-like structures in vitro and stimulated neo-angiogenesis in vivo, improving neurological outcomes in SCI mice (compared with the control group, BMS scores improved significantly in the hPMSCs-Exos group beginning 21 days post SCI, *p* < 0.05) [61]. Hypoxia-pretreated MSC-derived exosomes could significantly enhance angiogenesis and functional recovery by increasing VEGF expression via HIF-1α upregulation [62]. Additionally, Huang et al. [63] found that exosomes derived from miR-126-modified MSC significantly promoted angiogenesis post-SCI by inhibiting SPRED1 and PIK3R2. Another recent study demonstrated that ADSC-derived exosomes inhibit ferroptosis through the NRF2/SLC7A11/GPX4 pathway, promoting angiogenesis after SCI [64].

Preserving the integrity of the BSCB is critical to prevent secondary injury processes like edema, oxidative stress, and inflammation [60]. One study suggested that exosomes derived from BMSC improve the integrity of the BSCB by activating the NF-κB p65 signaling pathway, inhibiting pericyte migration, and thus promoting functional recovery after SCI [65]. On the other hand, BMSC-derived exosomes restored tight junctions among cells through the TIMP2/MMP pathway, mitigating the disruption of the BSCB and improving functional recovery after SCI [66]. Another recent study reported that RGD-CD146CD271-modified UCMSC-derived exosomes specifically target vascular endothelial cells at the site of injury, effectively inhibiting the expression of MLCK through miR-501-5p, and thus reducing the disruption of tight junctions, stabilizing the BSCB and ultimately promoting the recovery of neurological functions in SCI mice [19].

In summary, the above findings highlight the therapeutic potential of MSC-derived exosomes in promoting angiogenesis and maintaining BSCB integrity. However, a previous study has revealed differences in post-SCI angiogenesis rates even between rats and mice [67], suggesting potentially greater interspecies divergence in humans. Thus, future work should prioritize human-relevant models (e.g. primates), multi-omics analysis of endothelial junction dynamics, and clinically translatable exosome engineering strategies that integrate biomechanical cues.

#### Inhibition of apoptosis and promotion of nerve regeneration

Apoptosis, a critical mechanism for neuron and supporting cell loss following SCI, can be effectively minimized by inhibiting the cell death signaling pathway. This not only reduces neuron loss but also promotes nerve and axon regeneration, offering a promising strategy for SCI treatment. One study has shown that exosomes secreted by miR-126-modified BMSC can significantly reduce the expression of pro-apoptotic proteins Bax and cleaved caspase-3, while increasing anti-apoptotic Bcl-2 levels, effectively inhibiting apoptosis. These exosomes also enhance nerve regeneration by upregulating Nestin and SOX2 expression [63]. Another study confirmed that BMSC-derived exosomes could inhibit neuronal apoptosis by stimulating the Wnt/β-catenin pathway [92]. Several studies have also revealed that miR-21, contained in MSC-derived exosomes, exerts anti-apoptotic effects through various mechanisms [93–95]. While the anti-apoptotic effect of miR-21 has been increasingly recognized, further investigation and validation into its specific molecular pathways is urgently needed to advance our understanding. Moreover, exosomes from hypoxia-induced ADSC, enriched with miR-499-5p, have been found to target JNK3, thereby regulating the JNK3/c-Jun apoptosis pathway and reducing neuronal apoptosis after SCI [96]. This underscores the need for continued research into the specific molecular pathways of exosomes. In addition to preventing cell apoptosis, MSC-derived exosomes are key in promoting neural regeneration. For example, human PMSC-derived exosomes improve motor function after SCI by activating endogenous neural progenitor cells and neurogenesis, likely involving the activation of the MEK/ERK/CREB pathway [68]. Wang et al. [69] reported that miR-199a-3p/145-5p in UCMSC-derived exosomes targets the NGF/TrkA signaling pathway, inducing axon growth by increasing the level of TrkA ubiquitination. Besides, it was shown that let-7a-5p in MSC-derived exosomes promotes neuronal regeneration and improves neurological recovery in SCI rats by modulating the HMGA2/SMAD2 axis [70].

Although MSC-derived exosomes are the most extensively studied in the field of SCI and show significant therapeutic potential, particularly in inhibiting neuronal apoptosis, promoting neural regeneration and enhancing functional recovery, they face similar challenges as NPC-derived exosomes. Despite some preliminary guidance [37–39], the complex mechanisms underlying the effects of MSC-derived exosomes, coupled with the fact that researchers have often focused on narrow, specific areas of interest, have led to inconsistent results and a lack of replicability across studies.

### iPSC-derived exosomes

iPSCs, derived from reprogrammed somatic cells, have a differentiation potential similar to ESC without the associated ethical concerns, making them particularly promising for clinical applications. Recent research has increasingly focused on the use of iPSC-derived exosomes for treating SCI (Fig. 4 & Table 1). For example, miR-199b-5p in iPSC-derived exosomes has been found to activate the PI3K signaling pathway by regulating hepatocyte growth factor, facilitating macrophage polarization from M1 to M2, and thereby reducing inflammation and supporting SCI recovery [71]. Recent finding suggested that engineered iPSC-derived exosomes containing miR-23b, miR-21-5p, and miR-199b-5p could suppress neuronal inflammation triggered by LPS and IFN γ, significantly improving recovery after SCI [72].Another study highlighted that let-7b-5p, enriched in iPSC-derived exosomes, effectively reduces pyroptosis of microglia/macrophages, promoting axonal growth while also inhibiting the expression of the let-7b-5p target gene LRIG3 and modulating the PI3K/Akt pathway to mitigate oxidative stress and inflammatory damage [73].

Although research on iPSC-derived exosomes for SCI repair remains relatively limited, their potentially great application value has been preliminarily demonstrated. Such iPSC-derived exosomes not only possess strong neuroprotective effects but also carry growth-promoting factors and various miRNAs that support neuronal survival and regeneration. Thus, iPSC-derived exosomes are poised to play an increasing role in SCI treatment, warranting further exploration and deeper investigation.

### DPSC-derived exosomes

Dental-derived stem cell transplantation has expanded to using DPSC-derived exosomes, showing their potential in treating SCI. One study demonstrated that exosomes derived from DPSC could target the HMGB1/TLR4/MyD88/NF-κB pathway, effectively inhibiting TLR4, MyD88, and NF-κB expression during cerebral ischemia/reperfusion injury. This inhibition reduced pro-inflammatory cytokines such as IL-6, IL-1β, and TNF-α, highlighting DPSC-derived exosome’s protective role in brain injury repair and anti-inflammation [74]. Liu et al. [26] were the first to explore the therapeutic potential of DPSC-derived exosomes for SCI repair. Their findings showed that DPSC-derived exosomes reduce macrophage M1 polarization by inhibiting the ROS-MAPK-NFκB P65 signaling pathway, thereby decreasing inflammation and nerve damage while promoting functional recovery (LPS group vs. LPS + EXO group: 33.67 ± 1.79 vs. 21.43 ± 1.922, *p* < 0.05). This suggested that DPSC-derived exosomes can effectively modulate inflammation and mitigate nerve injury following SCI. What makes DPSC particularly attractive is their noninvasive extraction from discarded teeth without ethical concerns. Given these advantages, DPSC-derived exosomes possess promise as a safe and effective therapeutic strategy for clinical applications in treating SCI. Future studies may be able to turn to the use of DPSC-derived exosomes.

## From bench to bedside: bridging the translational gap

To date, only one clinical trial has been registered and reported for exosome-based SCI therapies [75, 76]. This trial marks a critical milestone in the translational journey, providing the first clinical evidence for the safety and potential efficacy of intrathecal allogeneic HUC-MSC-exosomes in complete subacute SCI patients. Hower, in this single-center phase I, small-sample, uncontrolled study, the patients received other interventions, such as rehabilitation, which precluded an accurate assessment of the clinical efficacy of exosomes, reflecting the nascent stage of the field [77]. While preclinical studies and the above phase I clinical trial have demonstrated the therapeutic potential of stem cell-derived exosomes in SCI models and patients, there are still many challenges ahead, and a long way to go in the future [39].

### Clinical challenges in exosome translation

#### Standardization of production and characterization

Current exosome isolation methods—ultracentrifugation (UC), size-exclusion chromatography (SEC), and polymer-based precipitation—vary significantly in yield, purity, and scalability [39, 78–80]. For instance, UC, the widely used method, often co-isolates protein aggregates, while SEC requires specialized equipment. These inconsistencies hinder reproducibility across studies and complicate regulatory approval. Emerging technologies, such as microfluidic sorting and immunoaffinity capture, show promise for high-purity exosome isolation but remain cost-prohibitive for large-scale clinical use. All of these issues need to be addressed to allow for repeatability and comparability between studies.

#### Optimization and solvent compatibility

Preclinical studies report exosome doses ranging from 20 μg to 1 mg/kg, with no consensus on optimal dosing. For example, intravenous doses of 100–200 μg in rodent models improved functional recovery, but translating these findings to humans requires careful scaling. Additionally, exosomes are often suspended in phosphate-buffered saline or plasma-mimicking solutions, yet the ideal solvent-to-exosome ratio remains undefined (Table 1), which is also a question that deserves to be explored in depth [6, 31].

#### Delivery route and bioavailability

Intravenous administration, though minimally invasive, results in low exosome accumulation at the injury site due to systemic distribution and rapid clearance [81]. Intranasal administration may be an effective option for the treatment of CNS disorders that require crossing the blood–brain barrier or BSCB. However, it is important to note that the bioavailability of intranasally administered drugs may be affected by a number of factors, including the physicochemical properties of the drug and its ability to be absorbed within the nasal cavity [82]. Localized approaches, such as intrathecal or intralesional injection, enhance bioavailability but require invasive procedures and precise dosing. For instance, studies using intraspinal exosome delivery in rats achieved significant neuroprotection, but local anatomical abnormalities in clinically injured patients highlighted technical complexities in clinical translation.

#### Dose target specificity and species differences

Preclinical models predominantly target pathways like NF-κB, TLR4, STK-4, GPX4 and so on (Fig. 4 & Table 1), yet human SCI pathology involves pathways or targets that may differ. Species-specific differences in exosome biodistribution and target engagement further complicate extrapolation to humans. For example, miR-146a-5p, effective in rodent microglial modulation, may exhibit altered efficacy in human immune cells. And single-cell sequencing and others may be used more often in the future in conjunction with exosomes for target selection in SCI therapy [83].

### Future directions

To bridge the translational gap, future efforts should focus on standardized protocols, targeted delivery systems or routines, personalized medicine, collaborative clinical trials, such as **e**stablishing consensus guidelines for exosome isolation, characterization, and dosing.

At the same time, it may be difficult to achieve good efficacy with exosomes alone. Rather, the combination of stem cell-derived exosomes with other approaches for the treatment of SCI may become a more favorable solution. Several studies have been conducted to deliver stem-cell derived exosomes for SCI treatment by using biomaterial scaffolds with favorable results. Localized delivery of an adhesive hydrogel to SCI lesions resulted in significant recovery of neurological function in SCI rats after 28 days (the treatment groups’ BBB score of 6.44 ± 1.64 was significantly superior to that of the blank groups’ 3.11 ± 2.13 (*p* < 0.05)) [84]. In another study, the preservation and controlled release of EV at the site of SCI for up to 56 days was effectively facilitated by the development of a special hydrogel that inhibited reactive fibrotic scarring, reduced inflammatory response, and facilitated myelin sheath regeneration and axonal regeneration, all of which synergistically significantly enhanced tissue repair and recovery of motor function after SCI [81]. A further study used methacrylate hydrogel (GelMA) as a supportive material and loads it with 3D-cultured MSC-derived exosomes to autonomously construct a micro-needle array patch (GelMA-MN@3D-Exo) for SCI repair. The patch significantly improved the retention and release of 3D-Exo at the injury site, reducing SCI-induced neuroinflammation and glial scar formation. [85]. In the latest study, GelMA hydrogel was used to encapsulate and load MSC-derived small extracellular vesicles (sEV) carrying berberine for SCI treatment. This formulation promoted nerve regeneration and axon growth and improved functional recovery by inhibiting local inflammation as well as fibroblast proliferation and migration [86]. In addition, exosomes derived from human menstrual blood stem cells combined with hyperbaric oxygen therapy significantly reduced pro-inflammatory cytokine levels [87]. Therefore, in the future, it may be possible to combine stem cell-derived exosomes with other biomaterials, drugs or hyperbaric oxygen therapy for the treatment of spinal cord injuries for optimal efficacy.

Moreover, tailoring exosome sources (e.g., BMSCs vs. iPSCs) and cargo (e.g., miRNA or protein enrichment) based on injury severity and patient-specific biomarkers [42, 88, 89] and expanding multi-center trials with standardized endpoints are also important factors to further advance the field.

## Summary and prospects

SCI remains a formidable challenge in regenerative medicine, but stem cell-derived exosomes offer a cell-free therapeutic strategy with multifaceted benefits, including neuroprotection, immunomodulation, and angiogenesis. Despite promising preclinical outcomes, clinical adoption is hindered by methodological variability in exosome production, incomplete mechanistic understanding, and unresolved safety concerns. Recent advances in exosome engineering and delivery systems, such as CRISPR-modified exosomes [91] or hydrogel encapsulation [81, 84, 85], hold potential to enhance therapeutic precision. However, large-scale clinical validation and regulatory alignment are imperative to transform these innovations into viable therapies. Collaborative efforts between researchers, clinicians, and regulatory bodies will be pivotal in overcoming these barriers, ultimately delivering hope for SCI patients worldwide.

## Data Availability

Not applicable.

## Abbreviations

SCI: Spinal cord injury
CNS: Central nervous system
ESC: Embryonic stem cells
iPSC: Induced pluripotent stem cells
MSC: Mesenchymal stem cells
NSC: Neural stem cells
miRNA, miR: MicroRNAs
mRNA: Messenger RNAs
BSCB: Blood-spinal cord barrier
TNF-α: Tumor necrosis factor-α
IL: Interleukin
ROS: Free radicals and reactive oxygen species
DPSC: Dental pulp stem cell
NGF: Nerve growth factor
Bcl-2: B cell lymphoma-2
ceRNA: Competing endogenous RNAs
VEGF: Vascular endothelial growth factor
AMSC: Amniotic mesenchymal stem cells
EV: Extracellular vesicles
ADSC: Adipose-derived mesenchymal stem cells
BMSC: Bone marrow mesenchymal stem cells
UCMSC: Umbilical cord mesenchymal stem cells
LPS: Lipopolysaccharide
PMSC: Placental mesenchymal stem cells
